# Blood metabolic signatures of hikikomori, pathological social withdrawal

**DOI:** 10.1080/19585969.2022.2046978

**Published:** 2022-06-01

**Authors:** Daiki Setoyama, Toshio Matsushima, Kohei Hayakawa, Tomohiro Nakao, Shigenobu Kanba, Dongchon Kang, Takahiro A. Kato

**Affiliations:** a Department of Clinical Chemistry and Laboratory Medicine, Graduate School of Medical Sciences, Kyushu University, Fukuoka, Japan; b Department of Neuropsychiatry, Graduate School of Medical Sciences, Kyushu University, Fukuoka, Japan

**Keywords:** Hikikomori, plasma metabolome, social isolation, COVID-19, acylcarnitine

## Abstract

**Background:**

A severe form of pathological social withdrawal, ‘hikikomori,’ has been acknowledged in Japan, spreading worldwide, and becoming a global health issue. The pathophysiology of hikikomori has not been clarified, and its biological traits remain unexplored.

**Methods:**

Drug-free patients with hikikomori (*n* = 42) and healthy controls (*n* = 41) were recruited. Psychological assessments for the severity of hikikomori and depression were conducted. Blood biochemical tests and plasma metabolome analysis were performed. Based on the integrated information, machine-learning models were created to discriminate cases of hikikomori from healthy controls, predict hikikomori severity, stratify the cases, and identify metabolic signatures that contribute to each model.

**Results:**

Long-chain acylcarnitine levels were remarkably higher in patients with hikikomori; bilirubin, arginine, ornithine, and serum arginase were significantly different in male patients with hikikomori. The discriminative random forest model was highly performant, exhibiting an area under the ROC curve of 0.854 (confidential interval = 0.648–1.000). To predict hikikomori severity, a partial least squares PLS-regression model was successfully created with high linearity and practical accuracy. In addition, blood serum uric acid and plasma cholesterol esters contributed to the stratification of cases.

**Conclusions:**

These findings reveal the blood metabolic signatures of hikikomori, which are key to elucidating the pathophysiology of hikikomori and also useful as an index for monitoring the treatment course for rehabilitation.

## Introduction

‘Hikikomori’, a severe form of pathological social withdrawal or social isolation that persists for more than 6 months and entails rarely leaving home, has long been observed in Japan (Kato et al. 2011, 2018, 2019). The prevalence of hikikomori is estimated to be 1.2% in the Japanese population (Koyama et al. 2010). Hikikomori is spreading worldwide and has become a global health issue, causing an enormous burden on society (Kato et al. 2012; Teo et al. 2015; Kato et al. 2018, 2016, 2019; Wu et al. 2019). Social and psychological factors are suggested as key causes of hikikomori (Kato et al. 2019, 2020; Nonaka and Sakai 2021), and the COVID-19 pandemic is suggested as a crucial risk factor for the rapid increase of hikikomori worldwide (Centre for the Mathematical Modelling of Infectious Diseases COVID-19 Working Group 2020; Kato et al. 2020; Yoshikawa et al. 2021). Comorbidity of psychiatric disorders, such as depression, schizophrenia, and social anxiety disorder is common (Teo et al. 2015; Kato et al. 2019, 2020). Increasing attention has recently been paid to biological factors that characterise mental illnesses (Miller et al. 2009; Kelley et al. 2021; Tamminga et al. 2021). Thus, while there may be biological underpinnings for hikikomori, they are not yet elucidated. Social isolation is a crucial issue worldwide, and its biological roots are particularly highlighted as a consequence of the COVID-19 pandemic (Hawkley and Cacioppo 2003; Koyama et al. 2021). We believe that biological research focussing on hikikomori has merit for clarifying the biological aspects of social isolation.

The discovery of many blood biomarkers has dramatically advanced the elucidation of the pathophysiology of psychiatric disorders (Sen et al. 2008; Gururajan et al. 2016). Regarding various physical diseases (diabetes, cancer, etc.), the physiological state can be predicted by monitoring changes in effective biomarkers in the blood (i.e., HbA1c). Similarly, the state or severity of hikikomori may be assessed if the biological traits that approximate hikikomori-related symptoms are discovered.

Ours and other groups have reported plasma metabolite biomarkers for major depressive disorder (MDD) using a mass-spectrometry-based omics approach (Setoyama et al. 2016, 2021, Kuwano et al. 2018). Furthermore, as patients with MDD exhibit various symptoms, such as depressive mood and suicidal ideation, the degree of which varies from patient to patient, there has been warning against collectively (homogeneously) studying MDD patients. Instead, heterogeneity should be taken into consideration (Goldberg 2011; Feczko and Fair 2020). In this regard, we recently observed that the diagnostic performance of blood biomarkers, such as tryptophan, was improved for a population stratified using the big-5 personality traits (Setoyama et al. 2021).

In this study, we performed blood biochemical tests and plasma metabolome analyses of serum/plasma from drug-free patients with hikikomori and age- and sex-matched healthy controls to identify biomarkers of hikikomori that characterise pathological social withdrawal tendencies. This is the first report to identify the blood biomarkers of drug-free individuals with hikikomori. We believe that the identification of hikikomori-related biomarkers will contribute not only to developing hikikomori support systems but also solving the present crucial social and psychological issues of social isolation during the global COVID-19 pandemic.

## Materials and methods

### Recruiting and sample collection

The present study was approved by the ethics committee of Kyushu University (IRB number: 2020-360) and was conducted in accordance with the Declaration of Helsinki. All participants were informed of the aims and methods of the present study, and that their participation was voluntary. Participants who agreed to participate in the study completed written informed consent and received a gift card incentive worth approximately US$25.

This study enrolled drug-free persons with hikikomori and healthy volunteers. All participants were Japanese (Asian). Age and sex were matched in all groups. All participants were recruited through the Mood Disorder/Hikikomori Clinic in the Department of Neuropsychiatry at Kyushu University Hospital and affiliated institutes, located in Fukuoka, southern Japan, which is the only hikikomori-specified research clinic in the world (Harding 2018).

#### Patients with hikikomori

We conducted an original semi-structured interview of patients with hikikomori. According to our proposed definition of hikikomori (pathological social withdrawal; Kato et al. 2018, 2020), the diagnosis of hikikomori in this study was defined as [a. physical isolation] marked social isolation and spending the majority of time at home (going out <4 days per week), [b. duration] continuous social isolation for ≥6 months, and [c. distress] significant functional impairment or distress associated with social isolation. Among the individuals who met the above definition of hikikomori, those who were not taking any psychotropic medications were included. Psychiatric comorbidities were assessed using the Japanese version of the Mini-International Neuropsychiatric Interview (M.I.N.I.) and Structured Clinical Interview for DSM-IV Axis I Disorders (SCID-I), a semi-structured psychiatric interview. All interviews were performed by trained psychiatrists or psychologists with extensive clinical experience and familiarity with the assessment tools.

#### Healthy controls

The above evaluations were conducted on healthy volunteers. Controls consisted of healthy volunteers who were not currently experiencing hikikomori symptoms or psychiatric conditions and were not taking any psychotropic medications.

### Psychological scales

We used the 25-item Hikikomori Questionnaire (HQ-25, score range 0–100), which is a self-rated assessment tool that was developed and validated recently to assess the severity of hikikomori, including three aspects of socialisation, isolation, and emotional support (Kato et al. 2020). According to our previous findings, depression and autism spectrum disorder (ASD) are often comorbid with hikikomori (Katsuki et al. 2020; Teo et al. 2020). The severity of depression was assessed with the Patients Health Questionaire-9 (PHQ-9; Kroenke et al. 2001; Arroll et al. 2010), the Beck Depression Inventory-II (BDI-II) by self-rated questionnaires (Kojima et al. 2002), and the 17-item Hamilton Depression Rating Scale (HAMD-17) using semi-structured interviews (Hamilton 1967; Furukawa et al. 2005). Autism spectrum tendencies were assessed with the Japanese version of the Autism-Spectrum Quotient (AQ-J; Wakabayashi et al. 2004).

Modern type depression (MTD) is an emerging condition in which premorbid personality is thought to differ from traditional melancholic-type depression in Japan (Kato et al. 2011, 2016). MTD is characterised by the occurrence of depressive symptoms mainly in stressful workplaces or school settings and a rapid disappearance of symptoms once patients leave these stressful situations. Hikikomori and MTD have similar tendencies, such as social avoidance; thus, we proposed that prolongation of MTD may lead to the occurrence of hikikomori, as a gateway disorder (Kato and Kanba 2017). To assess personality tendencies of MTD, we used our recently developed self-rated questionnaire, the 22-item Tarumi’s Modern-Type Depression Trait Scale (TACS-22, score range 0–88), which contains three factors: avoidance of social roles, complaints, and low self-esteem (Kato et al. 2019; Katsuki et al. 2020).

### Blood collection

Peripheral venous blood samples were collected between 10:00 and 15:00. The plasma and serum were immediately extracted and stored at –80 °C until analysis. Psychiatric and psychological evaluations were conducted on the day of blood collection.

### Serum arginase activity and nitric oxide (NO) measurement

Serum arginase activity and NO were measured using the QuantiChrom™ Arginase Assay Kit (BioAssay Systems, #DARG-100) and the QuantiChrom Nitric Oxide Assay Kit (BioAssay Systems, #D2NO-100), respectively, according to the manufacturer’s instructions.

## Results

We collected serum/plasma from drug-free patients with hikikomori (*N* = 42), mean age, 30.2 (SD = 8.2) years and their age-sex matched healthy controls (*N* = 41; Table 1). Twenty-four percent of the patients were diagnosed with MDD and the remaining 76% were subclinical for MDD. HAMD-17 and PHQ-9 scores were significantly higher among patients with hikikomori. In addition, modern-type depression traits (TACS-22), severity of depression (BDI-II), and autism tendencies (AQ-J) were also significantly higher among patients with hikikomori (Table 1).

**Table 1.** Participant demographics.

Characteristic	HC, *N* = 41^1^	Hikikomori, *N* = 42^1^	*p*-value^2^
MDD			<0.001
Negative	41 (100%)	32 (76%)	
Positive	0 (0%)	10 (24%)	
Sex (Male/Female)	25/16	25/17	>0.89
Age	31.1(6.1)	30.2(8.2)	0.58
Height	166.2(7.9)	163.7(7.8)	0.19
Weight	63.2(11.8)	61.8(14.0)	0.66
BMI	22.6(3.0)	23.0(4.9)	0.8
HAMD-17*	**1.0(2.3)**	**9.1(5.3)**	**<0.001**
PHQ-9*	**1.9(2.4)**	**11.3(6.8)**	**<0.001**
TACS-22*	**34.5(9.6)**	**47.3(11.8)**	**<0.001**
TACS-22_Avoidance	21.6(5.6)	22.4(5.8)	0.74
TACS-22_LowSE	9.0(3.2)	15.8(4.7)	<0.001
TACS-22_Complaint	3.8(3.0)	9.1(4.8)	<0.001
BDI-II*	**3.9(3.4)**	**24.5(13.2)**	**<0.001**
BDI-II_Affective	0.8(1.0)	4.6(2.9)	<0.001
BDI-II_Motivational	0.4(0.6)	2.6(1.7)	<0.001
BDI-II_Cognitive	0.4(0.7)	2.4(1.8)	<0.001
BDI-II_CD	0.8(1.0)	7.2(4.3)	<0.001
BDI-II_Behavioral	0.6(0.9)	4.2(2.9)	<0.001
BDI-II_PV	0.7(0.9)	2.2(1.8)	0.001
AQ*	**15.4(6.6)**	**25.4(7.3)**	**<0.001**
AQ_SS	2.8(2.7)	6.5(2.6)	<0.001
AQ_AS	3.4(1.6)	5.8(2.1)	<0.001
AQ_AD	3.6(2.2)	3.9(2.0)	0.68
AQ_CM	2.3(2.0)	4.6(2.4)	<0.001
AQ_IM	3.2(1.8)	4.6(2.1)	0.003
HQ-25*	**23.0(14.2)**	**67.7(18.5)**	**<0.001**
HQ-25_Socialisation	13.2(8.5)	30.6(9.0)	<0.001
HQ-25_Isolation	5.7(4.2)	22.9(6.3)	<0.001
HQ-25_ES	4.1(3.6)	13.7(6.6)	<0.001

^1^*n* (%); Mean(SD).

^2^Pearson’s Chi-squared test; Welch Two Sample *t*-test; Fisher’s exact test.

*Bold letters indicate the total score of each index.

**Figure 1.**
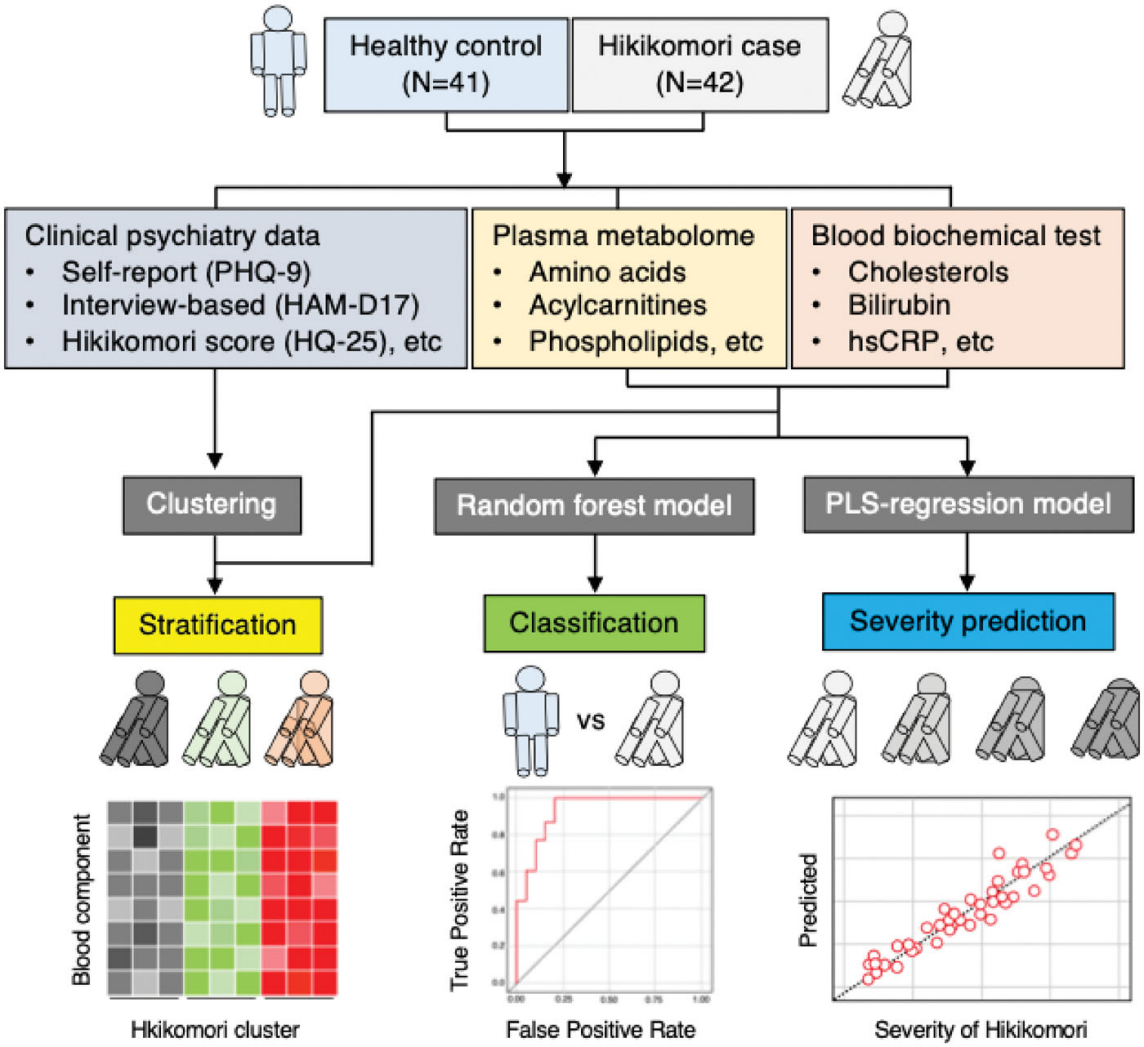
An overview of our research.

Blood biochemical tests and plasma metabolome analyses were performed. A total of 127 components of information were obtained (Table 2). Based on this information, integrated with clinical data, we sought metabolic signatures that contribute to discrimination, severity prediction, and stratification of cases, according to the research workflow (Figure 1).

**Table 2.** Summary of blood biochemical test and metabolome data.

Components	Healthy Controls (*n* = 41)^1^	Patients with Hikikomori (*n* = 42)^1^	*p*-value^2^
HDL.C	63(16)	61(14)	0.39
LDL.C	106(31)	127(42)	0.013
Total.C	170(32)	187(39)	0.027
FIB	228(38)	239(80)	0.44
FDP	0.12(0.55)	0.14(0.64)	>0.99
Total Bilirubin	0.8(0.4)	0.6(0.3)	0.004
Direct Bilirubin	0.26(0.11)	0.19(0.1)	0.012
Ratio of Bilirubin	0.5(0.3)	0.4(0.2)	0.007
Uric acid	5.2(1.4)	5.1(1.3)	0.93
hsCRP	654(1250)	793(2062)	0.71
Alanine	1037(403)	1182(341)	0.083
Arginine	51,346(31,538)	25,338(23,171)	<0.001
Asparagine	1776(366)	1791(557)	0.89
Aspartic acid	1114(345)	1174(363)	0.44
Citrulline	613,501(147,549)	561,635(172,096)	0.14
Creatine	165,835(54,564)	157,759(46,247)	0.47
Cystine	3036(2168)	1649(1775)	0.002
Glutamine	97,576(17,949)	96,825(14,885)	0.84
Glutamic acid	6689(1448)	6100(1101)	0.041
Hydroxyproline	6475(3133)	5820(3123)	0.34
Isoleucine	16,314(6451)	15,363(3960)	0.42
Leucine	22,268(9175)	20,200(5031)	0.21
Lysine	279,475(122,610)	249,511(116,314)	0.26
Methionine	4741(1439)	4006(1343)	0.018
Ornithine	361,159(185,011)	579,094(319,818)	<0.001
Phenylalanine	300,140(55,371)	274,499(43,596)	0.022
Proline	88,109(25,823)	104,856(59,013)	0.1
Serine	2111(496)	2200(918)	0.58
Taurine	60,874(16,619)	63,369(16,543)	0.5
Threonine	11,002(3342)	11,346(3324)	0.64
Tryptophan	628,450(113,946)	615,980(123,463)	0.63
Tyrosine	101,201(31,397)	85,419(21,983)	0.01
Valine	177,586(39,993)	172,954(28,363)	0.55
AC_C2:0	8769(6160)	9138(5794)	0.78
AC_C3:0	777(725)	1005(881)	0.2
AC_C4:0	563(523)	846(900)	0.084
AC_C5:0	455(563)	614(700)	0.26
AC_C3:DC	320(334)	353(372)	0.66
AC_C4:OH	294(278)	336(407)	0.58
AC_C6:0	743(448)	1262(1671)	0.058
AC_C4:DC	386(239)	443(252)	0.29
AC_C5:DC	395(219)	456(327)	0.32
AC_C8:1	15,217(6629)	14,191(8344)	0.54
AC_C8:0	12,204(7031)	19,418(24,067)	0.069
AC_C6:DC	282(109)	395(315)	0.033
AC_C9:0	3015(1138)	2750(1625)	0.39
AC_C10:2	4396(1860)	4307(2683)	0.86
AC_C10:1	30,504(15,302)	34,144(32,789)	0.52
AC_C10:0	47,853(27,080)	66,638(73,486)	0.13
AC_C10:OH	2432(1340)	2133(1350)	0.31
AC_C12:1	25,846(9573)	25,028(17,623)	0.79
AC_C12:0	19,106(7372)	22,124(17,136)	0.3
AC_C12:OH	1858(592)	1634(697)	0.12
AC_C14:2	17,969(7170)	17,170(12,683)	0.72
AC_C14:1	35,891(13,357)	36,212(27,282)	0.95
AC_C14:0	10,608(2560)	14,314(5542)	<0.001
AC_C14:OH	1823(585)	1847(829)	0.88
AC_C16:1	16,650(5710)	22,278(10,914)	0.004
AC_C16:0	52,566(16,743)	74,484(22,878)	<0.001
AC_C16:1OH	3300(715)	3724(1199)	0.054
AC_C16:OH	1443(512)	1616(639)	0.18
AC_C18:2	54,152(31,972)	98,696(46,648)	<0.001
AC_C18:1	79,666(43,510)	137,516(81,017)	<0.001
AC_C18:2OH	2169(557)	2485(574)	0.013
AC_C18:1OH	2008(510)	2297(1127)	0.14
AC_C18:OH	1224(385)	1350(348)	0.12
LPC_16:0	126,340(49,391)	153,125(48,812)	0.015
LPC_18:0	10,976(5305)	11,710(4143)	0.48
LPC_18:1	18,435(6407)	19,459(6346)	0.47
LPC_18:2	81,407(31,206)	77,605(28,363)	0.56
LPC_20:3	1976(1053)	2026(975)	0.82
LPC_20:4	16,606(7110)	15,764(6132)	0.57
LPC_20:5	6594(6156)	4886(3263)	0.12
LPC_22:6	6578(3400)	6031(2934)	0.44
PC_28:1	1634(1217)	1494(1315)	0.62
PC_30:0	5098(3340)	5846(4854)	0.42
PC_30:1	62,908(43,824)	66,422(57,301)	0.75
PC_30:2	3422(2471)	3706(3855)	0.69
PC_32:0	68,460(27,117)	68,459(35,397)	>0.99
PC_32:1	25,859(11,514)	29,573(17,556)	0.26
PC_32:2	4937(4756)	6749(7513)	0.19
PC_34:0	99,595(45,374)	108,182(67,563)	0.5
PC_34:1	1,092,711(495,597)	1,191,406(739,639)	0.48
PC_34:2	1,400,965(1,309,075)	1,468,986(1,407,794)	0.82
PC_34:3	7902(8181)	10,609(13,851)	0.28
PC_34:4	522(480)	716(1124)	0.31
PC_36:1	319,569(112,915)	327,641(147,198)	0.78
PC_36:2	1,792,206(649,366)	1,700,176(680,038)	0.53
PC_36:3	226,164(149,431)	256,024(190,455)	0.43
PC_36:4	477,734(402,968)	524,188(554,005)	0.66
PC_36:5	33,563(36,195)	56,715(129,592)	0.27
PC_38:1	2780(1822)	2444(1608)	0.38
PC_38:2	120,940(38,406)	124,738(44,872)	0.68
PC_38:3	200,924(73,251)	228,560(111,601)	0.19
PC_38:4	781,098(301,525)	711,718(395,633)	0.37
PC_38:5	112,188(83,000)	109,839(124,236)	0.92
PC_38:6	338,619(298,840)	388,828(405,709)	0.52
PC_40:1	950(407)	846(455)	0.27
PC_40:2	561(197)	534(292)	0.62
PC_40:3	2361(934)	2604(1437)	0.36
PC_40:4	18,664(6552)	20,657(11,668)	0.34
PC_40:5	65,229(32,072)	68,420(45,761)	0.71
PC_40:6	334,494(164,609)	332,576(194,790)	0.96
PC_40:7	15,603(12,586)	15,803(13,557)	0.94
PE_34:1	23,707(4095)	24,155(4407)	0.63
PE_34:2	3611(1082)	3911(1004)	0.19
PE_36:1	5938(1341)	5798(1476)	0.65
PE_36:2	59,882(8454)	62,781(10,955)	0.18
PE_36:3	916(794)	963(620)	0.76
PE_36:4	1933(1605)	2064(1710)	0.72
PE_38:4	10,156(4996)	9709(5198)	0.69
PE_38:5	1217(1025)	1139(977)	0.73
PE_38:6	4735(4138)	5096(4497)	0.7
CE_17:0	1951(896)	2080(1043)	0.55
CE_18:1	533(294)	512(293)	0.74
CE_18:2	3038(1065)	2936(1292)	0.7
CE_18:3	1445(626)	1444(612)	>0.99
CE_20:0	5220(1076)	4888(1511)	0.25
CE_20:1	11399(2974)	11,071(3376)	0.64
CE_20:2	4878(1270)	4404(1365)	0.11
TG_46:2	2731(2158)	3766(3312)	0.1
TG_46:3	675(215)	695(253)	0.7
TG_48:3	5013(2360)	5500(3261)	0.44
TG_50:2	205,464(134,673)	241,561(191,301)	0.32
TG_50:3	49,547(35,793)	54,265(43,494)	0.59
TG_52:2	1,114,558(586,713)	1,266,807(926,127)	0.37
TG_52:3	744,179(432,015)	775,286(533,412)	0.77

^1^Mean(SD); *n* (%).

^2^Welch Two Sample *t*-test; Fisher’s exact test.

The unit of each metabolite represents the mass spectrometric signal intensity (arbitrary unit, AU).

### Comparison of blood biochemical and plasma metabolome biomarker levels

**Figure 2.**
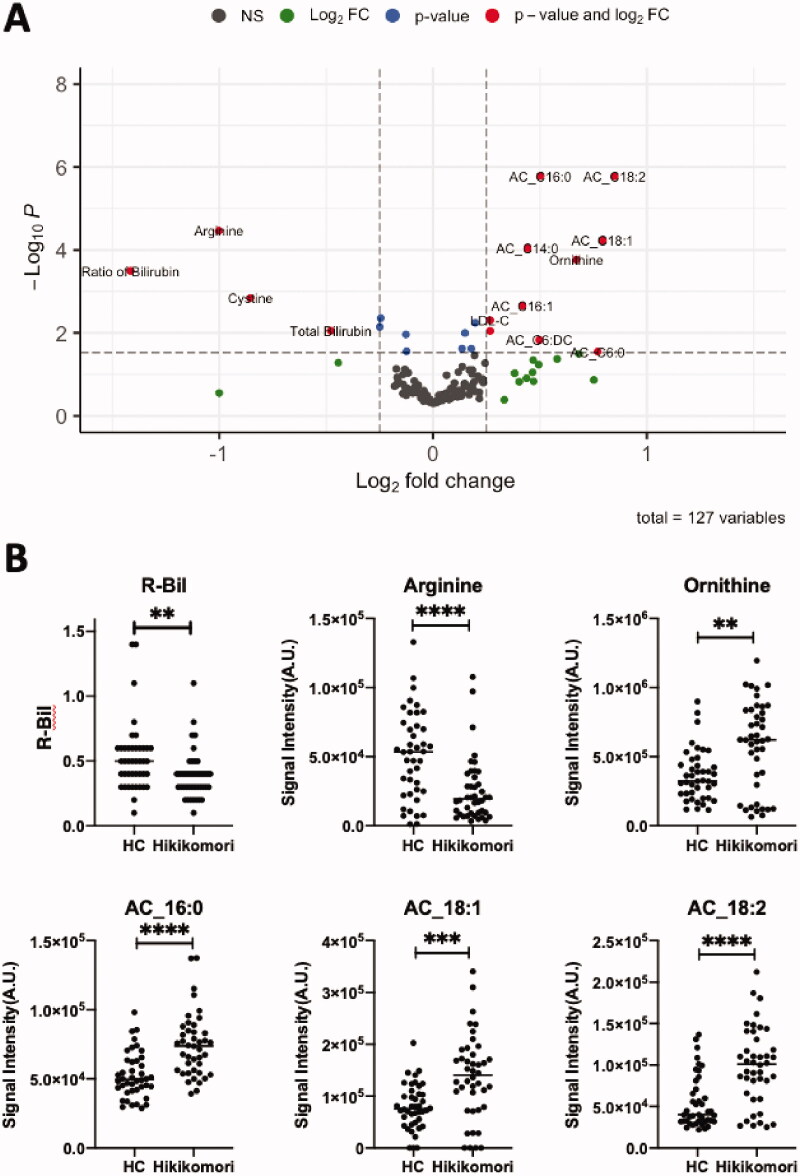
Differences in blood components between healthy controls and hikikomori patients. (A) Volcano plot showing significantly different blood components. The dotted line on the horizontal axis indicates *p* = 0.03. The two dotted lines on the vertical axis each indicate –0.25 and 0.25, respectively. AC refers to acylcarnitine, and after the underscore indicates the number of carbons and double bonds of the bound fatty acid. The blood components indicated by red dots indicate significant differences; blue dots indicate significant but small changes; green dots indicate changes but not significant; black dots indicate none of them. (B) Representative blood components. Black bars indicate medians. **p < 0.01; ***p < 0.001, ****p < 0.0001 (Welch two sample *t*-test).

Blood biochemical tests and plasma components were compared between groups (Table 2). Significantly different components were plotted in the volcano plot as individual dots (Figure 2(A)). The most prominent differences were observed in the ratio of bilirubin (R-Bil, a ratio of direct/indirect bilirubin), which was lower in patients with hikikomori. Long-chain acylcarnitines (AC_16: 0, AC_16: 1, AC_18: 1, AC_18.2) were higher in patients with hikikomori (Figure 2(A,B)). Amino acids, such as cystine, arginine, and ornithine, were remarkably different (Figure 2(B)). Total cholesterol and LDL cholesterol were slightly higher in patients with hikikomori, while there was no significant difference in phospholipids (Table 2).

### Machine-learning based hikikomori discrimination model

**Figure 3.**
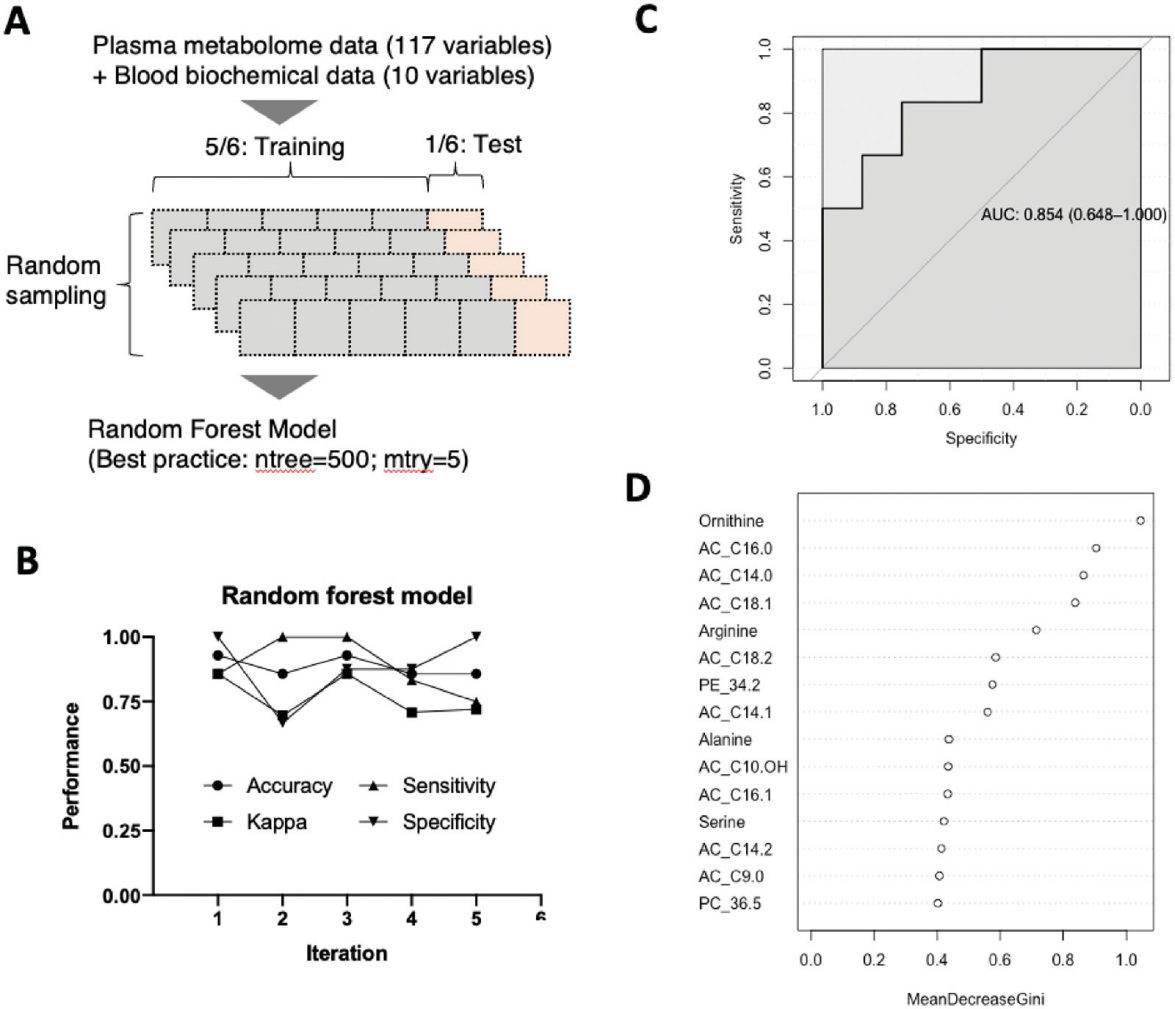
Machine-learning based hikikomori discrimination model. (A) Information of plasma metabolome and blood biochemical tests integrated, all data were divided into training and test data, of which five sets were created in an independent random sampling. With the parameters, ntree and mtry turned as 500 and 5, respectively, each random forest discrimination model was created. (B) Parameters to evaluate the performance of each iterated model. Kappa statistic represents adjusted accuracy that excludes the probability of discrimination by chance. (C) A receiver operating curve (ROC) displaying the performance of a representative discrimination model. The curve plots the sensitivity against the specificity for different cut points. Area under curves (AUCs) were calculated as 0.854 (95% CI = 0.648–1.00, *p* < 0.01)). (D) A variable importance plot of a representative discrimination model. The *x*-axis units on the plot indicate the mean decrease in Gini index. PE and PC stand for phosphatidylethanolamine and phosphatidylcholine, respectively.

We then created a random forest model to discriminate patients with hikikomori from healthy controls. All data were divided into six parts, of which five were used as training data for model creation and one was used as model verification test data (Figure 3(A)). To avoid division bias, random sampling was repeated five times to create a model for each dataset (Figure 3(A)). The discriminative models were highly performant on average, with a kappa value (an indicator of adjusted accuracy that excludes the probability of by-chance) above 0.7 (Figure 3(B), Supplemental Table S1). Because a kappa statistic over 0.6 is considered substantially significant (Cohen 1960), the performance of our discrimination model was considered practical. Supporting this, another performance indicator, the area under the receiver operating characteristic (ROC) curve (AUC) of the representative model was 0.854 (CI = 0.648–1.000; Figure 3C). The important blood components that contributed to the model are shown in Figure 3(D). Ornithine, arginine, and acylcarnitines (AC_16:0, AC_14:0, and AC_18:1) were the top five ranked components.

### Plasma arginase is another metabolic signature in male hikikomori cases

**Figure 4.**
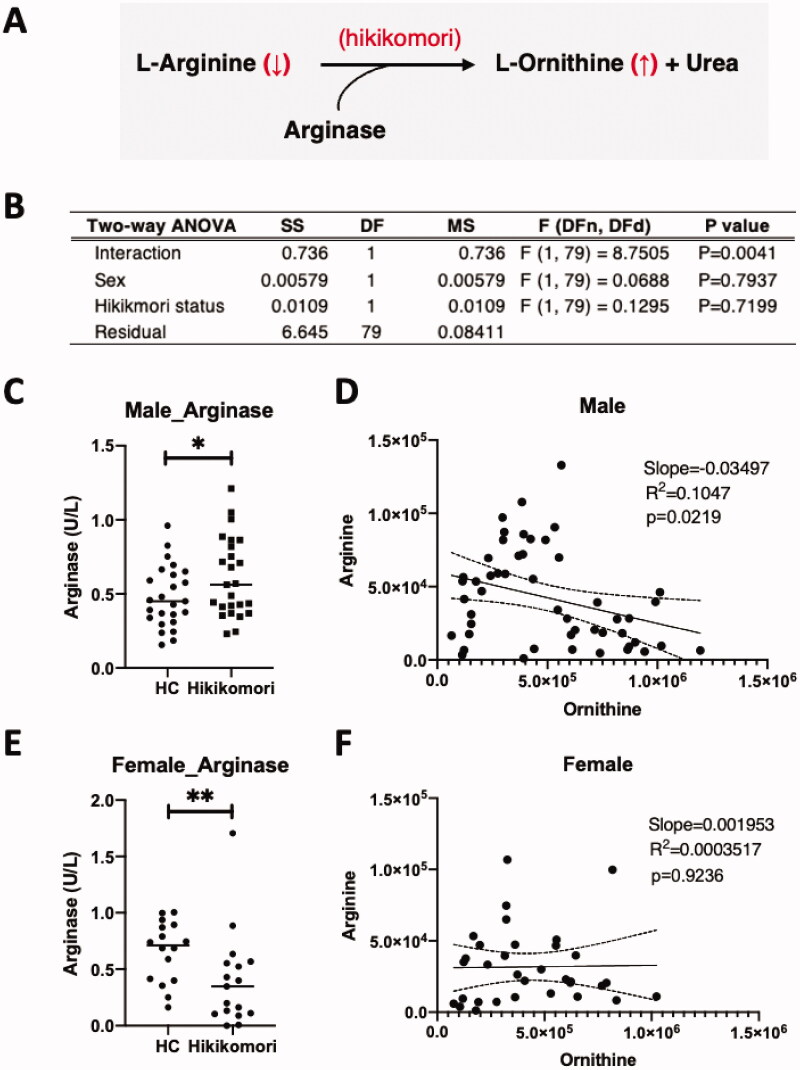
Another metabolic signature of serum arginase. (A) Enzymatic reaction of serum arginase that can explain the significant changes seen in patients with hikikomori. (B) Measurement results of serum arginase (Supplemental Figure S2B) were analysed by two-way analysis of variance (ANOVA), for which the two categorical factors were sex (male vs female) and group (HC vs hikikomori). (C) Comparison of serum arginase activity in males. *p < 0.05. (D) The relationship between arginine and ornithine in males’ plasma was analysed and fitted by linear regression showing with the 95% confidence bands in dot-line. (E) Comparison of serum arginase activity in female. **p < 0.01. (F) The relationship between arginine and ornithine in female plasma, as in (D).

Notably, plasma level of ornithine was higher and that of arginine was lower in hikikomori cases (Figure 2(B)), and both contributed to the discrimination model (Figure 3(D)). These findings prompted us to investigate the involvement of blood arginase, which produces ornithine using arginine as a substrate (Figure 4(A)). In fact, a mild negative correlation was observed between arginine and ornithine (Supplemental Figure S1(A)), while the activity of serum arginase did not differ significantly between the two groups (Supplemental Figure S2(B)). However, when the activity was stratified by sex and hikikomori status, the four-group test indicated significance (*p* = 0.00279; Supplemental Figure S1(C)). Furthermore, two-way ANOVA revealed significant interactions between sex and hikikomori status on serum arginase activity (Figure 4(B)). Post-hoc analysis indicated that the serum arginase activity of patients with hikikomori was significantly higher than controls, but only for male patients (Figure 4(C,E)). Consistently, blood levels of arginine (substrate) and ornithine (the product) were significantly different (Supplemental Table S2) and inversely correlated among males (Figure 4(D)), but not significantly correlated among females (Figure 4(F)). These findings strongly support the observation of increased serum arginase activity only in males. Alternatively, arginine is also metabolised by NO synthases (NOSs), but when NO_2_^–^/NO_3_^–^, which reflects the product NO, was measured, no significant sex difference was detected (Supplemental Figure S2(D)).

### Severity prediction model

Next, a partial least squares PLS-regression model was created to predict the severity of hikikomori (HQ-25) using biochemical tests and blood metabolome information (Figure 4(A)). The performance of the regression model was evaluated from the value of the root mean square of error (RMSE), demonstrating an average of 18.5, and a mean absolute error (MAE) of 15.5, respectively (Supplemental Table S3). Moreover, the average correlation coefficient was 0.8, indicating a highly linear prediction model. Blood components that contributed to this model include acylcarnitines (AC_C16:0, etc.) and amino acids (arginine, etc.), which appear similar to those that contribute to discriminating hikikomori groups from healthy controls (Figure 4(B)). In fact, AC_C16: 0 was significantly positively correlated with the HQ-25 score (Figure 4(C)), while arginine was negatively correlated (Figure 4(D)). These results strongly suggest that the severity of hikikomori can be predicted with practical accuracy using blood component information.

### Biomarkers for the stratification of hikikomori patients

Patients with hikikomori are considered a heterogeneous population, due to their diverse symptoms and social/personality backgrounds. Therefore, to identify blood components that contribute to the stratification of hikikomori populations, we performed principal component cluster analysis using clinical psychiatric data for all participants (Figure 6(A)). Interestingly, all participants were divided into three clusters. Cluster-1 was mainly included healthy controls; cluster-3 included patients with hikikomori. In contrast, cluster-2 contained half of the healthy controls and patients with hikikomori, respectively. Depression-related items (HAMD-17, PHQ-9, and BDI-II) were higher in patients with cluster-3 than in those with cluster-2 (Figure 6(B)). Moreover, given that 24% of patients in this study were diagnosed with MDD (Table 1) and many were sub-clinical for MDD, it is plausible that the patients in cluster-3 were those with MDD or were prone to depression. Based on this classification, we identified blood components that characterised each cluster of the hikikomori population (Figure 6(C)). Serum uric acid (UA) and some molecular species of cholesterol esters (CE_20:1, CE_18:3, and CE_20:2) were significantly different between the clusters (Figure 6(D)). There were also differences in the molecular species of triglycerides and phospholipids (Figure 6(C)), while no significant differences were observed in acylcarnitines or amino acids.

## Discussion

The prevalence of hikikomori is increasing worldwide, partly due to the global COVID-19 pandemic (Kato et al. 2020; Rooksby et al. 2020). The clinical features of hikikomori have gradually been clarified (Kato et al. 2018, 2019); however, objective evaluation using blood components has not yet been developed. The present study is the first to report a successful identification of blood metabolic signatures that are useful for discrimination, prediction of severity, and stratification of hikikomori (Figure 1).

### Bilirubin

We observed that all bilirubin-related test values (T-Bil, D-Bil, and R-Bil) were significantly lower in the blood of male patients with hikikomori (Figure 2, Table 2, and Supplemental Table S2). Bilirubin is a degradation product of red blood cells that is broken down in the liver and excreted in bile and is a marker of liver function in clinical practice (Jayanti et al. 2020). In contrast, bilirubin has an antioxidant capacity in the blood, suggesting that it is physiologically associated with various diseases other than liver disease (Rigato et al. 2005). Blood bilirubin levels are lower in patients with major depression (Peng et al. 2016) and urinary biopyrrins, oxidative metabolites of bilirubin, are higher in patients with various psychiatric disorders (Miyaoka et al. 2005). Interestingly, blood bilirubin levels are also lower in patients with seasonal affective disorder (SAD; Oren et al. 2002). A lack of sunlight is suggested to result in impaired circadian rhythm and cause SAD (Oren et al. 2013). Hikikomori is defined as ‘staying at home for at least 6 months’ (Kato et al. 2020), and thus a lack of sunshine may affect the circadian rhythm of patients with hikikomori and follow similar clinical courses as SAD (Oren et al. 2013). Therefore, treatment strategies that focus on the similarities between hikikomori and SAD are of great interest. Low bilirubin levels may be important state markers in the process of transition from a non-pathologically tendency to rarely leave home to hikikomori (pathological social withdrawal).

### Acylcarnitines

**Figure 5.**
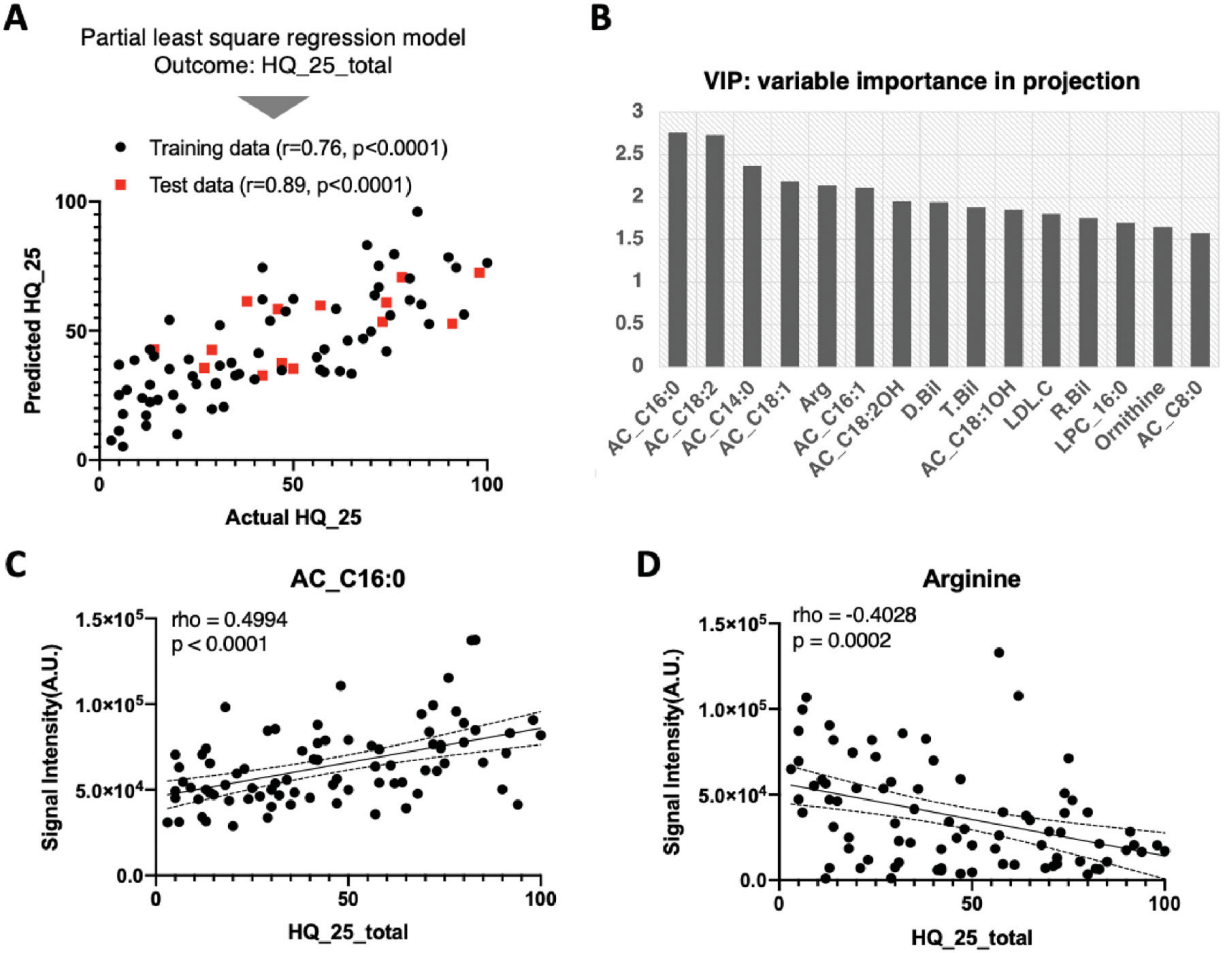
Machine-learning based model for the prediction of hikikomori severity (HQ-25). (A) PLS-regression model was created to predict HQ-25. In a representative model, actual HQ-25 scores and the predicted values are plotted; the training data are in black and the test data in red. (B) Variables’ importance to the projection (VIP) was visualised. AC: acylcarnitine; Arg: arginine; Bil: bilirubin; LDL: low-density lipoprotein cholesterol; LPC; lysophosphatidylcholine. (C) The relationship between palmitoylcarnitine (AC_C16:0) and HQ-25 was analysed by Spearman’s rank correlation and fitted by linear regression showing the 95% confidence bands in dot-line. (D) The relationship between arginine and HQ-25 was analysed as in (C).

Long-chain acylcarnitines were significantly higher in patients with hikikomori, regardless of sex, than controls (Figure 2 and Supplemental Table S2). We observed that these metabolites are important metabolic signatures with a high contribution in both discrimination and severity prediction models (Figures 3 and 5). Acylcarnitines are used as a substrate for mitochondrial β-oxidation and play an important role in the energy supply of the brain or physical function (Calabrese et al. 2006; Lum et al. 2011; Reuter and Evans 2012). Notably, in a rat model of depression, short- to long-chain acylcarnitines in the blood were elevated and then decreased with the administration of antidepressants (Chen et al. 2014). Moreover, acylcarnitines decrease when patients with depression take a selective serotonin reuptake inhibitor (SSRI; Ahmed et al. 2020). Therefore, patients with mood disorders seem to have elevated acylcarnitines in the blood (MahmoudianDehkordi et al. 2021), which may reflect impaired brain function. Interestingly, patients with hikikomori differ from patients with depression, in that only the long-chain acylcarnitines are elevated, which may also reflect the difference in metabolic utilisation of acylcarnitines in the brain between patients with hikikomori and those with depression.

**Figure 6.**
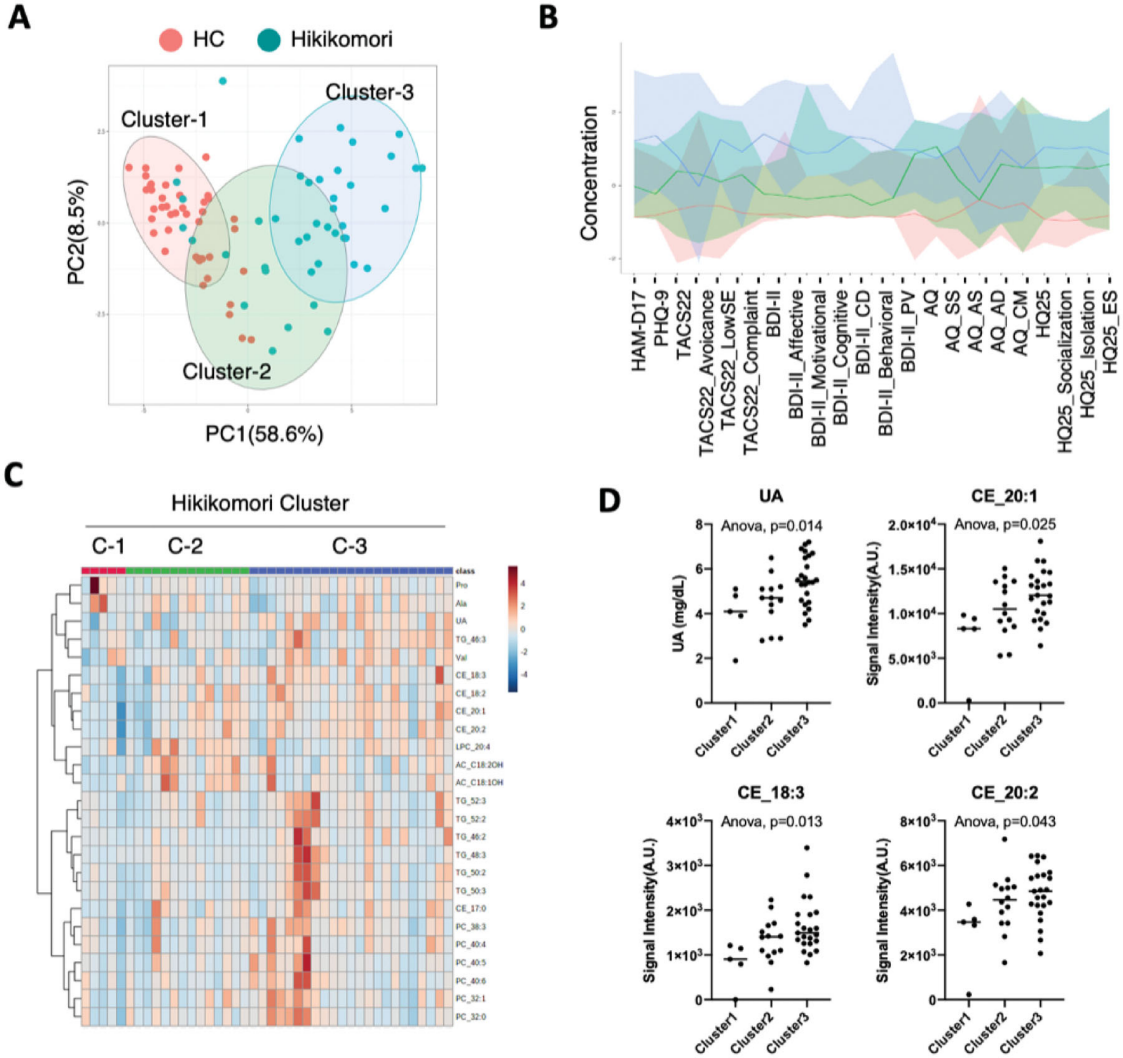
Stratification of patients with hikikomori. (A) Patients with hikikomori were analysed by principal component analysis for clustering information from all psychological scales, in which the first two components (PC1/PC2) accounted for 67.1% of the total variables. (B) Factor analysis showing the psychological scales that contribute to each cluster. Red, green, and blue lines indicate clusters 1, 2, and 3, respectively. (C) Cluster information was assigned to patients with hikikomori, supervised PLS discriminant analysis was performed, and the top 25 contributing blood components were shown on a heat map. Colour indicator shows relative intensity in plasma. (D) Representative blood components that characterise each cluster were shown with the ANOVA results. A black bar in each graph means median. UA: uric acid; CE: cholesterol ester.

### Arginine/ornithine/arginase

We observed that plasma arginine and ornithine are important metabolic signatures with a high contribution to the discrimination and severity prediction models (Figures 2, 3, and 5). Interestingly, among patients with hikikomori for males, but not females, plasma arginine was significantly lower, ornithine was higher and the two were negatively correlated with each other, suggesting that ornithine is partly regulated by the activation of serum arginase (Figure 4, Supplemental Tables S1 and S2). In this regard, serum arginase is associated with depression severity (Elgun and Kumbasar 2000; Caldwell et al. 2018) and urinary 8-OHdG, an indicator of oxidative stress (Ogino et al. 2013). Although it is unclear why arginine, ornithine, and arginase are involved only for male patients with hikikomori, it should be noted that our results clearly revealed a sex difference in blood components that reflect the hikikomori state.

Given that arginine is also a source of NO production, a decrease in arginine in hikikomori plasma might be reflected in the increased activity of NO synthase (NOS; Ceylan et al. 2011; Ogino et al. 2017). However, there seems to be no significant difference in serum NO, as evaluated from the values of serum NO_2_^–^/NO_3_^–^ (Supplemental Figure S1(D)), suggesting that NOS activity is unaffected. However, the decrease in plasma arginine inevitably affects the ability to produce NO in the blood (Bronte and Zanovello 2005; Caldwell et al. 2018). In fact, serum arginase is recognised as the major arginine-depleting enzyme (Riess et al. 2018) and its activation carries the risk of interfering with various NO-related physiological phenomena. Thus, arginine auxotrophy, a supplement therapy, may be effective in improving various symptoms in male patients with hikikomori.

We previously reported a pilot study focussing on blood biomarkers of hikikomori for the first time with a smaller sample, suggesting that uric acid (UA) and HDL-C levels in men and FDP and hsCRP in women are correlated with avoidant personality scores, the most common psychiatric comorbidity of hikikomori (Hayakawa et al. 2018). However, in this study, we did not detect a significant difference in UA and HDL-C in males or hsCRP in females (Table 2). This discrepancy between studies is probably due to the presence or absence of medication: plasma/serum samples in this study were all drug-free, whereas some of the patients in the previous study were taking medication (Hayakawa et al. 2018). It is highly possible that some blood components, including clinical tests, are greatly affected by medication, as acylcarnitines change with medication in patients with depression (Mood Disorders Precision Medicine Consortium ¥(MDPMC¥) 2020) and rat models of depression (Chen et al. 2014). To verify this, it is necessary to evaluate the blood components of patients with hikikomori while considering the presence or absence of medication.

### Clinical significance of hikikomori biomarkers

Here, we created a random forest model to discriminate patients with hikikomori based on integrated data from blood metabolome and clinical test values. A severity prediction model with practical accuracy was created. In addition, after stratifying patients with hikikomori based on clinical data, we identified that the above blood components contributed to their categorisation. Altogether, our findings not only advance the understanding of the blood biomarkers to discriminate patients with hikikomori from healthy controls but also elucidate the heterogeneity of hikikomori.

We revealed that hikikomori can be stratified by the presence or absence of depressive symptoms. Each patient with hikikomori may experience psychiatric issues. Depression-targeting treatment strategies may be the first choice for hikikomori sufferers with depressive symptoms. Patients with hikikomori without depressive symptoms may require other approaches, such as social support, rather than targeting depressive symptoms.

The support system for hikikomori is warranted, given our previous and present understanding of the pathophysiology (Kato et al. 2020). We expect that the biomarkers of hikikomori presented here will contribute to establishing biology-based hikikomori support systems to objectively evaluate and classify the conditions of hikikomori and then determine the appropriate treatment choice. Therefore, stratification of patients with hikikomori by biomarkers would allow tailor-made support for patients. In the initial stage of hikikomori support, we propose classifying biotypes by measuring biomarkers. Some hikikomori cases seem to be significantly affected by biological factors; therefore, biological approaches, such as nutritional therapy, may be useful for these individuals.

### Limitations and future perspectives

This study has some limitations. First, although the sample size was larger than that in our previous study (Hayakawa et al. 2018), it was still relatively small. However, all the participants in this study were drug-free, which is a significant advantage. Second, this study did not compare the presence or absence of exercise habits. Although there was no significant difference in BMI between the patients with hikikomori and the healthy controls (*p* = 0.8), future studies should evaluate physical activity levels. Third, blood biomarkers, such as amino acids and proteins, identified in the present study might be influenced by diet and protein intake. Thus, these factors should be measured as covariate factors. In a small study with multiple biomarkers, there is a high likelihood of false-positive findings. Finally, this study was conducted only in Japan. Currently, hikikomori is spreading worldwide; thus, international investigations should be conducted to understand the similarities and differences between patients with hikikomori in Japan and other countries.

Despite these limitations, one of the most significant advantages of the present study is that all the data were collected based on the world’s only research clinic specialising in hikikomori. This is the first report to identify the blood biomarkers of drug-free individuals with hikikomori. Acylcarnitines are related to both male and female patients with hikikomori. Bilirubin, arginine, ornithine, and arginase were related, especially among male patients with hikikomori. Further investigations in different countries are needed to validate these findings. Biological interventions should be developed to support patients with hikikomori.

## Supplementary Material

Supplemental Material

## References

[CIT0001] Ahmed AT, Mood Disorders Precision Medicine Consortium (MDPMC), MahmoudianDehkordi S, Bhattacharyya S, Arnold M, Liu D, Neavin D, Moseley MA, Thompson JW, Williams LSJ, Louie G, et al. 2020. Acylcarnitine metabolomic profiles inform clinically-defined major depressive phenotypes. J Affect Disord. 264:90–97. 32056779 10.1016/j.jad.2019.11.122 PMC7024064

[CIT0002] Akio W, Yoshikuni T, Simon B-C, Sally W. 2004. The Autism-Spectrum Quotient (AQ) Japanese version: evidence from high-functioning clinical group and normal adults. Shinrigaku Kenkyu: Jap J Psychol. 75:78–84. 10.4992/jjpsy.75.78 15724518

[CIT0003] Arroll B, Goodyear-Smith F, Crengle S, Gunn J, Kerse N, Fishman T, Falloon K, Hatcher S. 2010. Validation of PHQ-2 and PHQ-9 to screen for major depression in the primary care population. Ann Fam Med. 8(4):348–353. 20644190 10.1370/afm.1139 PMC2906530

[CIT0004] Bronte V, Zanovello P. 2005. Regulation of immune responses by L-arginine metabolism. Nat Rev Immunol. 5(8):641–654. 16056256 10.1038/nri1668

[CIT0005] Calabrese V, Giuffrida Stella AM, Calvani M, Butterfield DA. 2006. Acetylcarnitine and cellular stress response: roles in nutritional redox homeostasis and regulation of longevity genes. J Nutr Biochem. 17(2):73–88. 16413418 10.1016/j.jnutbio.2005.03.027

[CIT0006] Caldwell RW, Rodriguez PC, Toque HA, Narayanan SP, Caldwell RB. 2018. Arginase: a multifaceted enzyme important in health and disease. Physiol Rev. 98(2):641–665. 29412048 10.1152/physrev.00037.2016 PMC5966718

[CIT0007] Ceylan NÖ, Çimenci İG, Kılınçaslan A, Bülbül F, Savaş HA. 2011. Alterations in plasma nitric oxide level and arginase activity during the treatment of bipolar depressive episode. Psychiatry Behav Sci. 1:1–6.25379211

[CIT0008] Chen S, Wei C, Gao P, Kong H, Jia Z, Hu C, Dai W, Wu Y, Xu G. 2014. Effect of Allium macrostemon on a rat model of depression studied by using plasma lipid and acylcarnitine profiles from liquid chromatography/mass spectrometry. J Pharm Biomed Anal. 89:122–129. 24284228 10.1016/j.jpba.2013.10.045

[CIT0009] Cohen J. 1960. A coefficient of agreement for nominal scales. Educ Psychol Meas. 20:37–46. DOI: 101177/001316446002000104.

[CIT0010] Elgun S, Kumbasar H. 2000. Increased serum arginase activity in depressed patients. Prog Neuro-Psychopharmacol Biol Psychiatry. 24(2):227–232. 10.1016/s0278-5846(99)00100-1 10800745

[CIT0011] Feczko E, Fair DA. 2020. Methods and challenges for assessing heterogeneity. Biol Psychiatry. 88(1):9–17. 32386742 10.1016/j.biopsych.2020.02.015 PMC8404882

[CIT0012] Furukawa TA, Streiner DL, Azuma H, Higuchi T, Kamijima K, Kanba S, Ozaki N, Aoba A, Murasaki M, Miura S, et al. 2005. Cross-cultural equivalence in depression assessment: Japan-Europe-North American study. Acta Psychiatr Scand. 112(4):279–285. 16156835 10.1111/j.1600-0447.2005.00587.x

[CIT0013] Goldberg D. 2011. The heterogeneity of ‘major depression’. World Psychiatry. 10(3):226–228. 21991283 10.1002/j.2051-5545.2011.tb00061.x PMC3188778

[CIT0014] Gururajan A, Clarke G, Dinan TG, Cryan JF. 2016. Molecular biomarkers of depression. Neurosci Biobehav Rev. 64:101–133. 26906761 10.1016/j.neubiorev.2016.02.011

[CIT0015] Hamilton MAX. 1967. Development of a rating scale for primary depressive illness. Br J Soc Clin Psychol. 6(4):278–296. 6080235 10.1111/j.2044-8260.1967.tb00530.x

[CIT0016] Harding C. 2018. Hikikomori. Lancet Psychiatry. 5(1):28–29. 29277213 10.1016/S2215-0366(17)30491-1

[CIT0017] Hawkley LC, Cacioppo JT. 2003. Loneliness and pathways to disease. Brain Behav Immun. 17(1):98–105. 10.1016/s0889-1591(02)00073-9 12615193

[CIT0018] Hayakawa K, Kato TA, Watabe M, Teo AR, Horikawa H, Kuwano N, Shimokawa N, Sato-Kasai M, Kubo H, Ohgidani M, et al. 2018. Blood biomarkers of Hikikomori, a severe social withdrawal syndrome. Sci Rep. 8(1):2884. 29440704 10.1038/s41598-018-21260-w PMC5811600

[CIT0019] Hellewell J, Abbott S, Gimma A, Bosse NI, Jarvis CI, Russell TW, Munday JD, Kucharski AJ, Edmunds WJ, Funk S, Centre for the Mathematical Modelling of Infectious Diseases COVID-19 Working Group, et al. 2020. Feasibility of controlling COVID-19 outbreaks by isolation of cases and contacts. Lancet Glob Health. 8(4):e488–e496. 32119825 10.1016/S2214-109X(20)30074-7 PMC7097845

[CIT0020] Jayanti S, Moretti R, Tiribelli C, Gazzin S. 2020. Bilirubin and inflammation in neurodegenerative and other neurological diseases. Neuroimmunol Neuroinflamm. 7:92–108.

[CIT0021] Kato TA, Kanba S. 2017. Modern-type depression as an ‘adjustment’ disorder in japan: the intersection of collectivistic society encountering an individualistic performance-based system. Am J Psychiatry. 174(11):1051–1053. 29088934 10.1176/appi.ajp.2017.17010059

[CIT0022] Kato TA, Kanba S, Teo AR. 2016. A 39-year-old ‘adultolescent’: understanding social withdrawal in Japan. Am J Psychiatry. 173(2):112–114. 26844793 10.1176/appi.ajp.2015.15081034 PMC5573246

[CIT0023] Kato TA, Kanba S, Teo AR. 2018. Hikikomori: experience in Japan and international relevance. World Psychiatry. 17(1):105–106. 29352535 10.1002/wps.20497 PMC5775123

[CIT0024] Kato TA, Kanba S, Teo AR. 2019. Hikikomori : multidimensional understanding, assessment, and future international perspectives. Psychiatry Clin Neurosci. 73(8):427–440. 31148350 10.1111/pcn.12895

[CIT0025] Kato TA, Kanba S, Teo AR. 2020. Defining pathological social withdrawal: proposed diagnostic criteria for hikikomori. World Psychiatry. 19(1):116–117. 31922682 10.1002/wps.20705 PMC6953582

[CIT0026] Kato TA, Sartorius N, Shinfuku N. 2020. Forced social isolation due to COVID-19 and consequent mental health problems: Lessons from hikikomori. Psychiatry Clin Neurosci. 74(9):506–507. 32654336 10.1111/pcn.13112 PMC7404367

[CIT0027] Kato TA, Shinfuku N, Sartorius N, Kanba S. 2011. Are Japan’s hikikomori and depression in young people spreading abroad? Lancet. 378(9796):1070. 21924990 10.1016/S0140-6736(11)61475-X

[CIT0028] Kato TA, Shinfuku N, Tateno M. 2020. Internet society, internet addiction, and pathological social withdrawal: The chicken and egg dilemma for internet addiction and hikikomori. Curr Opin Psychiatry. 33(3):264–270. 32101902 10.1097/YCO.0000000000000601

[CIT0029] Kato TA, Hashimoto R, Hayakawa K, Kubo H, Watabe M, Teo AR, Kanba S. 2016. Multidimensional anatomy of ‘modern type depression’ in Japan: a proposal for a different diagnostic approach to depression beyond the DSM-5. Psychiatry Clin Neurosci. 70(1):7–23. 26350304 10.1111/pcn.12360 PMC5560068

[CIT0030] Kato TA, Katsuki R, Kubo H, Shimokawa N, Sato-Kasai M, Hayakawa K, Kuwano N, Umene-Nakano W, Tateno M, Setoyama D, et al. 2019. Development and validation of the 22-item Tarumi’s modern-type depression trait scale: avoidance of social roles, complaint, and low self-esteem (TACS-22). Psychiatry Clin Neurosci. 73(8):448–457. 30900331 10.1111/pcn.12842 PMC6850625

[CIT0031] Kato TA, Shinfuku N, Fujisawa D, Tateno M, Ishida T, Akiyama T, Sartorius N, Teo AR, Choi TY, Wand APF, et al. 2011. Introducing the concept of modern depression in Japan; an international case vignette survey. J Affect Disord. 135(1-3):66–76. 21782250 10.1016/j.jad.2011.06.030

[CIT0032] Kato TA, Tateno M, Shinfuku N, Fujisawa D, Teo AR, Sartorius N, Akiyama T, Ishida T, Choi TY, Balhara YPS, et al. 2012. Does the ‘hikikomori’ syndrome of social withdrawal exist outside Japan? A preliminary international investigation. Soc Psychiatry Psychiatr Epidemiol. 47(7):1061–1075. 21706238 10.1007/s00127-011-0411-7 PMC4909153

[CIT0033] Katsuki R, Tateno M, Kubo H, Kurahara K, Hayakawa K, Kuwano N, Kanba S, Kato TA. 2020. Autism spectrum conditions in hikikomori: a pilot case-control study. Psychiatry Clin Neurosci. 74(12):652–658. 32940406 10.1111/pcn.13154 PMC7756345

[CIT0034] Kelley ME, Choi KS, Rajendra JK, Craighead WE, Rakofsky JJ, Dunlop BW, Mayberg HS. 2021. Establishing evidence for clinical utility of a neuroimaging biomarker in major depressive disorder: prospective testing and implementation challenges. Biol Psychiatry. 90(4):236–242. 33896622 10.1016/j.biopsych.2021.02.966 PMC8324510

[CIT0035] Kojima M, Furukawa TA, Takahashi H, Kawai M, Nagaya T, Tokudome S. 2002. Cross-cultural validation of the Beck Depression Inventory-II in Japan. Psychiatry Res. 110(3):291–299. 12127479 10.1016/s0165-1781(02)00106-3

[CIT0036] Koyama A, Miyake Y, Kawakami N, Tsuchiya M, Tachimori H, Takeshima T., World Mental Health Japan Survey Group, 2002-2006. 2010. Lifetime prevalence, psychiatric comorbidity and demographic correlates of ‘hikikomori’ in a community population in Japan. Psychiatry Res. 176(1):69–74. 20074814 10.1016/j.psychres.2008.10.019

[CIT0037] Koyama Y, Nawa N, Yamaoka Y, Nishimura H, Sonoda S, Kuramochi J, Miyazaki Y, Fujiwara T. 2021. Interplay between social isolation and loneliness and chronic systemic inflammation during the COVID-19 pandemic in Japan: results from U-CORONA study. Brain Behav Immun. 94:51–59. 33705870 10.1016/j.bbi.2021.03.007 PMC7939973

[CIT0038] Kroenke K, Spitzer RL, Williams JBW. 2001. The PHQ-9: validity of a brief depression severity measure. J Gen Intern Med. 16(9):606–606. 11556941 10.1046/j.1525-1497.2001.016009606.x PMC1495268

[CIT0039] Kuwano N, Kato TA, Setoyama D, Sato-Kasai M, Shimokawa N, Hayakawa K, Ohgidani M, Sagata N, Kubo H, Kishimoto J, et al. 2018. Tryptophan-kynurenine and lipid related metabolites as blood biomarkers for first-episode drug-naïve patients with major depressive disorder: An exploratory pilot case-control study. J Affect Disord. 231:74–82. 29454180 10.1016/j.jad.2018.01.014

[CIT0040] Lum H, Sloane R, Huffman KM, Kraus VB, Thompson DK, Kraus WE, Bain JR, Stevens R, Pieper CF, Taylor GA, et al. 2011. Plasma acylcarnitines are associated with physical performance in elderly men. J Gerontol A Biol Sci Med Sci. 66A(5):548–553. 10.1093/gerona/glr006 PMC3074959 21367961

[CIT0041] MahmoudianDehkordi S, Ahmed AT, Bhattacharyya S, Han X, Baillie RA, Arnold M, Skime MK, John-Williams LS, Moseley MA, Thompson JW, The Mood Disorders Precision Medicine Consortium (MDPMC), et al. 2021. Alterations in acylcarnitines, amines, and lipids inform about the mechanism of action of citalopram/escitalopram in major depression. Transl Psychiatry. 11(1):153. 33654056 10.1038/s41398-020-01097-6 PMC7925685

[CIT0042] Miller AH, Maletic V, Raison CL. 2009. Inflammation and its discontents: The role of cytokines in the pathophysiology of major depression. Biol Psychiatry. 65(9):732–741. 19150053 10.1016/j.biopsych.2008.11.029 PMC2680424

[CIT0043] Miyaoka T, Yasukawa R, Yasuda H, Shimizu M, Mizuno S, Sukegawa T, Inagaki T, Horiguchi J. 2005. Urinary excretion of biopyrrins, oxidative metabolites of bilirubin, increases in patients with psychiatric disorders. Eur Neuropsychopharmacol. 15(3):249–252. 15820412 10.1016/j.euroneuro.2004.11.002

[CIT0044] Nonaka S, Sakai M. 2021. Psychological factors associated with social withdrawal (Hikikomori). Psychiatry Invest. 18(5):463–470. 10.30773/pi.2021.0050 PMC8169334 34053211

[CIT0045] Ogino K, Ito T, Eguchi E, Nagaoka K. 2017. Association of arginase I or nitric oxide-related factors with job strain in healthy workers. PLoS ONE. 12(4):e0175696. 28403218 10.1371/journal.pone.0175696 PMC5389831

[CIT0046] Ogino K, Murakami I, Wang D-H, Tsukiyama Y, Takahashi H, Kubo M, Sakano N, Setiawan H, Bando M, Ohmoto Y, et al. 2013. Evaluation of serum arginase I as an oxidative stress biomarker in a healthy Japanese population using a newly established ELISA. Clin Biochem. 46(16-17):1717–1722. 24005081 10.1016/j.clinbiochem.2013.08.012

[CIT0047] Oren DA, Desan PH, Boutros N, Anand A, Charney DS. 2002. Effects of light on low nocturnal bilirubin in winter depression: a preliminary report. Biol Psychiatry. 51(5):422–425. 11904137 10.1016/s0006-3223(01)01254-9

[CIT0048] Oren DA, Koziorowski M, Desan PH. 2013. SAD and the not-so-single photoreceptors. Am J Psychiatry. 170(12):1403–1412. 23929223 10.1176/appi.ajp.2013.13010111

[CIT0049] Peng YF, Xiang Y, Wei YS. 2016. The significance of routine biochemical markers in patients with major depressive disorder. Sci Rep. 6:34402. 27683078 10.1038/srep34402 PMC5041142

[CIT0050] Reuter SE, Evans AM. 2012. Carnitine and acylcarnitines: Pharmacokinetic, pharmacological and clinical aspects. Clin Pharmacokinet. 51(9):553–572. 22804748 10.1007/BF03261931

[CIT0051] Riess C, Shokraie F, Classen CF, Kreikemeyer B, Fiedler T, Junghanss C, Maletzki C. 2018. Arginine-depleting enzymes – an increasingly recognized treatment strategy for therapy-refractory malignancies. Cell Physiol Biochem. 51(2):854–870. 30466103 10.1159/000495382

[CIT0052] Rigato I, Ostrow JD, Tiribelli C. 2005. Bilirubin and the risk of common non-hepatic diseases. Trends Mol Med. 11(6):277–283. 15949769 10.1016/j.molmed.2005.04.008

[CIT0053] Rooksby M, Furuhashi T, McLeod HJ. 2020. Hikikomori: a hidden mental health need following the COVID-19 pandemic. World Psychiatry. 19(3):399–400. 32931118 10.1002/wps.20804 PMC7491622

[CIT0054] Sen S, Duman R, Sanacora G. 2008. Serum brain-derived neurotrophic factor, depression, and antidepressant medications: meta-analyses and implications. Biol Psychiatry. 64(6):527–532. 18571629 10.1016/j.biopsych.2008.05.005 PMC2597158

[CIT0055] Setoyama D, Kato TA, Hashimoto R, Kunugi H, Hattori K, Hayakawa K, Sato-Kasai M, Shimokawa N, Kaneko S, Yoshida S, et al. 2016. Plasma metabolites predict severity of depression and suicidal ideation in psychiatric patients – a multicenter Pilot analysis. PLoS ONE. 11(12):e0165267. 27984586 10.1371/journal.pone.0165267 PMC5161310

[CIT0056] Setoyama D, Yoshino A, Takamura M, Okada G, Iwata M, Tsunetomi K, Ohgidani M, Kuwano N, Yoshimoto J, Okamoto Y, et al. 2021. Personality classification enhances blood metabolome analysis and biotyping for major depressive disorders: two-species investigation. J Affect Disord. 279:20–30. 33038697 10.1016/j.jad.2020.09.118

[CIT0057] Tamminga CA, Clementz BA, Pearlson G, Keshavan M, Gershon ES, Ivleva EI, McDowell J, Meda SA, Keedy S, Calhoun VD, et al. 2021. Biotyping in psychosis: using multiple computational approaches with one data set. Neuropsychopharmacology. 46(1):143–155. 32979849 10.1038/s41386-020-00849-8 PMC7689458

[CIT0058] Teo AR, Fetters MD, Stufflebam K, Tateno M, Balhara Y, Choi TY, Kanba S, Mathews CA, Kato TA. 2015. Identification of the hikikomori syndrome of social withdrawal: Psychosocial features and treatment preferences in four countries. Int J Soc Psychiatry. 61(1):64–72. 24869848 10.1177/0020764014535758 PMC5573567

[CIT0059] Teo AR, Nelson S, Strange W, Kubo H, Katsuki R, Kurahara K, Kanba S, Kato TA. 2020. Social withdrawal in major depressive disorder: a case-control study of hikikomori in japan. J Affect Disord. 274:1142–1146. 32663943 10.1016/j.jad.2020.06.011

[CIT0060] Teo AR, Stufflebam K, Saha S, Fetters MD, Tateno M, Kanba S, Kato TA. 2015. Psychopathology associated with social withdrawal: idiopathic and comorbid presentations. Psychiatry Res. 228(1):182–183. 25977071 10.1016/j.psychres.2015.04.033

[CIT0061] Wu AFW, Ooi J, Wong PWC, Catmur C, Lau JYF. 2019. Evidence of pathological social withdrawal in non-Asian countries: a global health problem? Lancet Psychiatry. 6(3):195–196. 30798886 10.1016/S2215-0366(18)30428-0

[CIT0062] Yoshikawa Y, Kumazaki H, Kato TA. 2021. Future perspectives of robot psychiatry: can communication robots assist psychiatric evaluation in the COVID-19 pandemic era? Curr Opin Psychiatry. 34(3):277–286. 33560019 10.1097/YCO.0000000000000692

